# Shunting Inhibition Controls the Gain Modulation Mediated by Asynchronous Neurotransmitter Release in Early Development

**DOI:** 10.1371/journal.pcbi.1000973

**Published:** 2010-11-04

**Authors:** Vladislav Volman, Herbert Levine, Terrence J. Sejnowski

**Affiliations:** 1 Center for Theoretical Biological Physics, University of California at San Diego, La Jolla, California, United States of America; 2 Computational Neurobiology Laboratory, The Salk Institute for Biological Studies, La Jolla, California, United States of America; 3 Howard Hughes Medical Institute, The Salk Institute for Biological Studies, La Jolla, California, United States of America; 4 Division of Biological Sciences, University of California at San Diego, La Jolla, California, United States of America; Université Paris Descartes, Centre National de la Recherche Scientifique, France

## Abstract

The sensitivity of a neuron to its input can be modulated in several ways. Changes in the slope of the neuronal input-output curve depend on factors such as shunting inhibition, background noise, frequency-dependent synaptic excitation, and balanced excitation and inhibition. However, in early development GABAergic interneurons are excitatory and other mechanisms such as asynchronous transmitter release might contribute to regulating neuronal sensitivity. We modeled both phasic and asynchronous synaptic transmission in early development to study the impact of activity-dependent noise and short-term plasticity on the synaptic gain. Asynchronous release decreased or increased the gain depending on the membrane conductance. In the high shunt regime, excitatory input due to asynchronous release was divisive, whereas in the low shunt regime it had a nearly multiplicative effect on the firing rate. In addition, sensitivity to correlated inputs was influenced by shunting and asynchronous release in opposite ways. Thus, asynchronous release can regulate the information flow at synapses and its impact can be flexibly modulated by the membrane conductance.

## Author Summary

Computation in a single neuron is regulated by gain control – the change in the sensitivity of a neuron to different patterns of stimulation. Hence, it is important to understand the different mechanisms underlying gain modulation. Earlier work focused on gain modulation in mature networks with functional connectivity; however, the mechanisms of gain control in the developing brain are still unclear. We show here that asynchronous release of neurotransmitter, a form of synaptic “noise” that is strongly expressed in synapses of the developing brain, and short-term synaptic plasticity can efficiently modulate the gain of the synapse without a priori assumptions about network's connectivity. Asynchronous release depends on the activity-dependent accumulation of synaptic calcium. We show that changes in asynchronous release can have either a divisive or multiplicative effect on the gain, depending on the state of the neuronal membrane conductance. Thus, activity-dependent synaptic “noise” can regulate the information flow at synapses, and its impact on the neuron is flexibly modulated by neuronal membrane conductance.

## Introduction

Gain control of synapses regulates the flow of information through neural circuits [1]. The neurophysiological mechanisms that modulate neuronal transfer functions have been the focus of several studies [2]–[6]. In particular, background noise and shunting inhibition have a divisive effect on the gain curve of a neuron [4], [5]. In contrast, presynaptic short-term depression extends the dynamic range over which a neuron can discriminate between different levels of afferent activity, and therefore represents another way to control the neuronal output [2], [7].

Another mechanism of gain modulation that has been extensively studied relies on “balanced inputs” [3], [8]. When the afferent excitation and inhibition are increased while maintaining a constant average membrane potential, the increase in the amplitude of the fluctuations leads to an increase in neuronal firing rate. However, control of the neuronal firing rate by modulating the balance between excitation and inhibition requires strict coordination between activities of upstream excitatory and inhibitory neurons, a condition that may be difficult to maintain [9]. This is especially true in the early developmental stages before the architecture of the network has been established. Thus, there may be alternative ways to achieve neuronal gain modulation.

Previous studies have assumed that short-term synaptic plasticity and noise act independently of one another to affect the input-output curve of a neuron. This assumption holds for the “phasic release” of neurotransmitter from a presynaptic terminal that occurs shortly after the arrival of an action potential. Although most of the fast communication between neurons is through fast phasic release, many central synapses have an additional, asynchronous component of transmitter release in response to stimulation [10]–[16]. Asynchronous release lasts for several hundred milliseconds following a synaptic stimulus, and is believed to occur due to the build-up of residual presynaptic calcium that enhances the probability of vesicular release [10], [17]. The extent of asynchronous release at a given time reflects the “history” of synaptic stimulation over hundreds of milliseconds. Asynchronous release is often considered a form of “noise” but should instead be seen as a form of slow, activity-dependent synaptic signaling. Several studies have suggested different roles for asynchronous release, including modulation of synaptic transmission [11], [12], [18], extension of the postsynaptic spike window [15] and the control of recurrent network dynamics [14], [19]. Asynchronous release may be dominant during the early stages of synaptic development, thus making it a prime candidate for gain modulation in the absence of tightly balanced excitation and inhibition.

We used a computational modeling approach to investigate the possible effects of asynchronous release on the modulation of synaptic gain, with specific focus on the early developmental period (rat post-natal days 20–30) before afferent inhibition is fully established [20]. We show here that the impact of asynchronous release critically depends on the level of shunting inhibition. In the low shunt regime, neuronal response is determined by the mean level of synaptic current, and a change in the level of asynchronous release leads to an approximately multiplicative modulation of the firing rate. In a high membrane conductance regime, asynchronous release acts to divisively modulate synaptic gain. Thus, shunting inhibition can vary the influence of asynchronous release over a wide range, from amplification to reduction. Correlated stimuli were also differentially processed by shunting inhibition and asynchronous release, therefore underscoring the potential importance of asynchronous release in network dynamics.

## Results

### Synchronized neuronal firing can explain measured post-synaptic response

**Figure 1:**
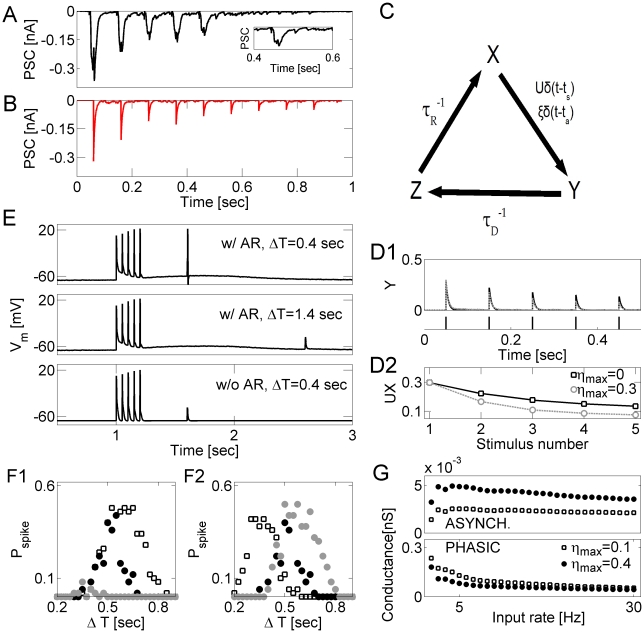
Asynchronous release modulates the spike generation window and attenuates the strength of phasic synaptic transmission. ***A*** Post-synaptic current recorded under rhythmic stimulation of a cultured hippocampal neuron by another neuron. Note that the significant level of asynchronous release (increase in the current level relative to 0) develops already after the first pulse and persists long after the last stimulus. ***B*** Postsynaptic current generated by model synapses (vesicular model, Methods) in response to rhythmic and synchronous stimulation at 10Hz. ***C*** Diagram of synaptic model. Transitions from the recovered (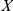) to active (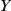) state are possible either via evoked (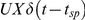) or asynchronous (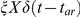) release. ***D*** Response of model synapse to rhythmic stimulation at 10 Hz: 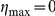 (solid black); 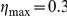 (dashed gray) (***D1***). ***D2***: the strength of phasic synaptic response, plotted vs. the stimulus number, for the model synapse with (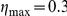, gray circles) and without (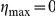, black squares) asynchronous release. ***E*** Modulation of spike time window by asynchronous release in model synapses. The probability to generate spike in response to a test stimulus depends on the time 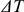 since the end of conditioning stimulation, and on the level of asynchronous release at model synapses. ***F*** Probability for spike generation, plotted vs. the time 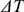 from the end of conditioning stimulation. Left panel shows the dependence of 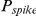
on the frequency of conditioning stimulation (number of stimuli is the same across all conditions, 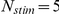): 5 Hz (gray circles), 10 Hz (black circles) and 20 Hz (squares). Right panel shows the dependence of 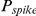 on the number of conditioning stimuli (frequency of stimulation is the same across all conditions, 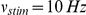): 3 stimuli (squares), 5 stimuli (black circles), 7 stimuli (gray circles). Data points are averages over 100 realizations. ***G*** Different components of synaptic conductance plotted vs. the rate of synaptic stimulation. The model synapse was stimulated by Poisson spike trains at 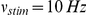. Top panel: conductance due to asynchronous component of synaptic transmission. Bottom panel: conductance due to phasic component of synaptic transmission. Open squares: 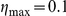. Closed circles: 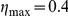.

Our goal was to understand the role of asynchronous release in modulating the gain of neurons. To accomplish this, we constructed a computational model that could capture some of the most salient features present in experimental data from hippocampal cell culture [12]. An example of this type of data is presented in Figure 1A. Although it is expected that at most one vesicle per stimulus was released from each hippocampal synapse in culture [21], [22], nonetheless the response exhibited “analog” behavior in that there was both depression in the phasic release component and a small but clearly detectable asynchronous release. From this, we concluded that vesicles were released synchronously from many sites immediately following the action potential and incorporated synchronous synaptic activation in our model, as described below.

To show how synchronization can lead to this type of experimental data, we first built a computational model of a synaptic terminal which featured a small number of vesicles that could be stochastically released either as a phasic response to synaptic stimulation or in an asynchronous manner (Methods). Rhythmic and coordinated stimulation of model synapses (Figure 1B) revealed the onset of synaptic depression, in qualitative agreement with the aforementioned experimental results from hippocampal cultures (Figure 1A). Crucially, the data could be explained with an extremely small (1%) level of assumed synchronization of release at different synapses. When asynchronous release was added to the model synapse, the response was further reduced (compared to the case without asynchronous release), because, according to the Equations 10–13 in Methods, both evoked and asynchronous releases were drawn from the same pool of resource [23]. Thus, by virtue of its competition with phasic release, the asynchronous release enhanced the effects of synaptic short-term depression [16], [24].

Since long-term simulation studies of our detailed synaptic model became computationally demanding for large (thousands) numbers of afferents, we developed a reduced and computationally much more efficient model of synaptic transmission that still incorporated both phasic and asynchronous components of release (Methods, also schematic in Figure 1C). This reduction is possible due to the fact that input to a specific neuron is equal to be a sum over many (∼30) jointly activated synapses. This type of model was originally introduced in an earlier study of the emergence and sustenance of rhythmic reverberations [19], which included the details of how this model was matched to experimental findings. The response of this reduced model to rhythmic synaptic stimulation was qualitatively similar to the response of the more detailed model (Figure 1D). The reduced model was thereafter used to investigate the impact of asynchronous release and shunting conductance on neuronal gain. We have also checked that the main conclusions obtained with the reduced model (namely, the dual shunting-dependent effect of AR on input-output curve) can also be reproduced with the more detailed synaptic model (Supplementary Figure S1).

### Asynchronous transmitter release modulates the time window for spike generation

Asynchronous release can modulate neuronal spiking in a time window that is dependent on the prior activity of synaptic afferents. Figure 1E illustrates how asynchronous release due to earlier synaptic activity can condition neuronal sensitivity to later stimuli. A conditioning set of 30 model synapses was synchronously and rhythmically stimulated by 5 pulses at 20 Hz. At time 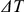 following the last preconditioning pulse, a test pulse was synchronously delivered to two additional (fully relaxed) model synapses. In the presence of asynchronous release due to an earlier, conditioning, stimulation, the test stimulus generated a somatic spike; blockade of the asynchronous release eliminated the spike (Figure 1E, bottom panel). As shown in Figure 1F, the probability of generating a somatic spike in response to a weak test stimulus depended both on the time 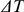 since the conditioning and on the earlier spike pattern (characterized here by the frequency and number of spikes).

The extent of asynchronous release at model synapses is determined by the availability of synaptic resource, which is reduced, and by the level of residual calcium, which builds up in the course of prolonged synaptic stimulation. For higher-frequency stimulation, which both depleted synaptic resource and led to a significant build up of residual calcium, the probability peaked later because it took longer for the synapse to recover and generate a sufficient level of asynchronous release. In contrast, when the stimulation rate approached the rate of residual calcium clearance, the level of asynchronous release at model synapses was low, in which case there was a low probability of generating a spike in response to a test stimulus (Figure 1F, left panel). Stimulation by a larger number of preconditioning pulses (while keeping the rate of stimulation constant) resulted in the shift of 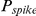 to the right (Figure 1F, right panel).

To further investigate the effects of asynchronous release on synaptic transmission we stimulated the model synapse with Poisson inputs at different rates and compared the spike-averaged peak conductance generated by the phasic component of release with the time-averaged conductance generated by the asynchronous component (Figure 1G). The asynchronous component of synaptic conductance was averaged over the window 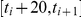 of only those inter-spike intervals that satisfied 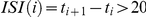, where 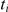 is the time of *i*-th spike that arrives at the synapse (time is measured in milliseconds). This conservative estimate ensured that the effects of conductance due to *i*-th phasic release are negligible. In Figure 1G, synaptic conductance due to the phasic mode of release was higher for lower stimulation rates, but decreased significantly for higher stimulation rates due to short-term synaptic depression. Stronger asynchronous release increased the conductance due to the asynchronous component and further reduced the peak phasic conductance.

These results suggest that asynchronous release can modulate how a neuron responds to synaptic inputs, but exactly how this occurs depends on the pattern of earlier synaptic activity. In what follows, we examine how this type of modulation contributes to synaptic gain control and how it depends on the intrinsic properties of the neuron, such as the membrane conductance.

### Asynchronous transmission accentuates gain modulation by short-term depression

How does the “activity-coupled “noise” introduced by asynchronous transmitter release affect the transfer characteristics of a neuron, and, more generally, how does short-term synaptic plasticity affect synaptic gain control? Central synapses have heterogeneous mixtures of facilitation and depression in responses to natural stimuli [25]. We first explored the changes in neuronal response that were caused by changes in the onset of, and recovery from, synaptic depression. These included changes in the recovery time from presynaptic short-term depression, 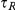, and the strength of phasic synaptic transmission, We first studied a model neuron in the low conductance regime (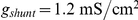).

**Figure 2:**
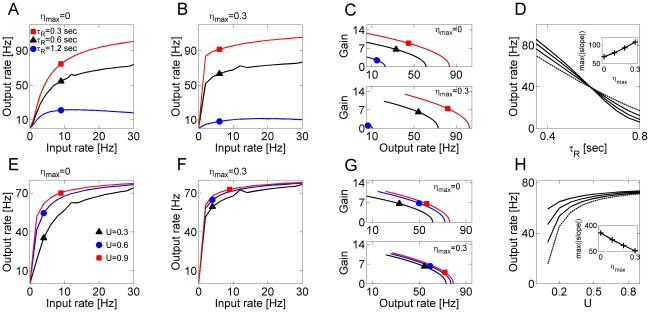
Gain control by short-term presynaptic depression. ***A*** Slower recovery from synaptic depression leads to a reduction and faster saturation of output firing rate: 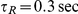 (red squares); 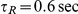 (black triangles); 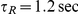 (blue circles). ***B*** An addition of asynchronous component (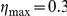) to synaptic transmission accentuates the depression-induced difference in neuronal gain. Symbols are the same as in ***A***. ***C*** Gains (defined as described in Text) plotted vs. the output rate, for the different scenarios shown in ***A,B***. Top panel: model synapses without asynchronous component of release. Bottom panel: model synapses with asynchronous release (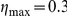). ***D*** Firing rate of a model neuron, plotted vs. the recovery time from synaptic depression, for Poisson input stimulation at 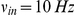. Dashed line: results obtained for a neuron driven by model synapses with no asynchronous release. Solid lines: 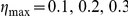. Inset: maximal absolute value of 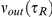 slope, plotted vs. the level of asynchronous release at model synapses. ***E*** Output firing rate vs. the input rate for different values of phasic coupling strength (as captured by 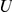): 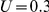 (black triangles); 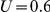 (blue circles); 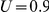 (red squares). ***F*** Output firing rate vs. the input rate for different values of phasic coupling strength (symbols are the same as in ***E***), but with the asynchronous release (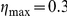) added to model synapses. ***G*** Gains plotted vs. the output rate, for the different cases considered in ***E,F***. Top panel: model synapses without asynchronous component of release. Bottom panel: model synapses with asynchronous release (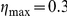). ***H*** Output firing rate plotted vs. the strength of phasic release, for Poisson input stimulation at 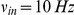. Dashed line: results obtained for a neuron driven by model synapses with no asynchronous release. Solid lines: 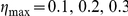. Inset: maximal absolute value of 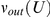 slope, plotted vs. the level of asynchronous release at model synapses.

In Figure 2A, with no asynchronous release (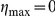), increasing the value of recovery time from presynaptic depression 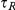 consistently decreased the output firing for high input rates and had a nearly divisive effect for lower input frequencies. When a relatively strong asynchronous component (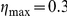) was added to the model for synaptic transmission (as in Figure 2B), a comparison of input-output curves for different recovery times revealed that, in this parameter regime, the effects of asynchronous release on the input-output curve depended on the rate at which model synapses recovered from depression. With an asynchronous component, the output rate for a model neuron with quickly recovering afferent synapses (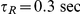) increased for all input rates, whereas the firing rate of a neuron with more slowly recovering afferents (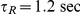) decreased following the same manipulation (Figures 2A,B). When plotted vs. the averaged recovery time, the output rate of a model neuron was always a monotonically decreasing function of 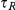 (Figure 2D, for fixed input rate of 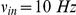), but the slope of the relation was controlled by the level of asynchronous release at the model synapses (inset of Figure 2D). Higher levels of asynchronous release resulted in a steeper 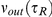 curve. Thus, asynchronous release of neurotransmitter in our model accentuated depression-related modulation of neuronal gain.

In Figure 2C the gains are plotted as a function of output rates (from Figures 2A,B) to examine the effects of recovery from depression. These gains were computed by taking the derivatives of the best second-order polynomial fits to the data of firing rates in Figures 2A,B (Chance et al., 2002). Note that in Figure 2C an additive shift in the abscissa of an input-output curve would have no effect, an upward shift of an input-output curve would cause translation along the abscissa, and a change in gain would result in the translation along the ordinate.

Another way to alter synaptic depression is by modulating 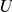, the strength of the phasic synaptic transmission. Figures 2E,F illustrate the effect of 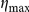 and 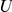 on the input-output function. For 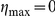, different strengths of phasic release gave rise to distinct input-output curves; however, with a relatively strong asynchronous component, the effect of phasic transmission became less pronounced (Figure 2F). The observation that changing the value of 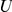 did not significantly affect the response of a neuron in the presence of asynchronous release means that, *in this parametric regime*, the fluctuations in the synaptic signal that arise due to the phasic release are not likely to be a decisive factor for spike generation. Rather, as is explained below, the synaptic gain was determined by the average level of synaptic current, which was in turn affected by asynchronous release. In contrast, with increased membrane conductance spike generation was driven by the presence of strong events in phasic transmission and 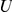 was influential (results not shown). These differences are also evident in Figure 2H, which shows the dependence of output rate (for a fixed input rate) on the value of 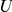 for different levels of asynchronous release. Without asynchronous release, the output rate critically depended on the strength of phasic transmission, but the addition of an asynchronous component saturated the dependence (Figure 2H). Figure 2G shows further that varying 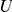 had little effect on the gain in the presence of asynchronous release.

### Membrane conductance modulates the effect of asynchronous release on synaptic gain

Membrane conductance strongly affects the excitability of the cell and shapes neuronal responses to excitatory afferents [4], [26], [27]. In particular, increasing the membrane conductance (shunting) reduces neuronal excitability and shifts the neuron from integrating weak perturbations at low shunt values to detecting coincidences between strong inputs at high shunt values. There is a similar shift in the mode of processing from phasic synaptic transmission, in which the neuron responds with high amplitude and a fast time-scale, to asynchronous transmission characterized by a low amplitude and a slow time-scale.

**Figure 3:**
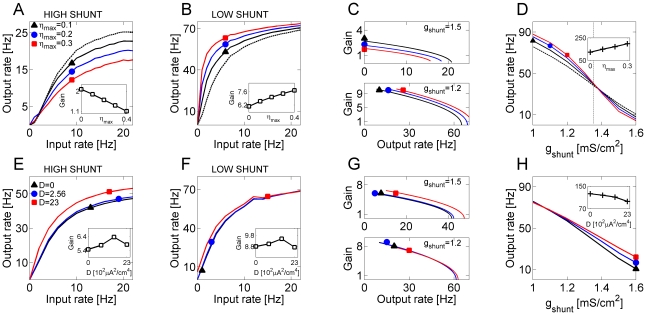
Membrane conductance defines the modulating action of asynchronous release on neuronal transfer function. ***A*** Transfer properties of a model neuron in high conductance (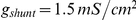) regime: 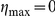 (dashed line); 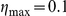 (black triangles); 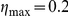 (blue circles); 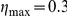 (red squares). Inset: gain (averaged slope of input-output curve) plotted vs. the level of asynchronous release. ***B*** Transfer properties of a model neuron in low conductance (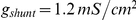) regime. Symbols are the same ones as in ***A***. Inset: gain (averaged slope of input-output curve) plotted vs. the level of asynchronous release. ***C*** Gains, plotted vs. the output rate, for different scenarios shown in ***A,B***. Top panel: gains for a model neuron in high conductance regime. Bottom panel: gains for a model neuron in low conductance regime. ***D*** Output rate plotted vs. membrane conductance, for Poisson stimulation at 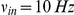. Symbols are the same as in ***A***. Inset: maximal absolute value of 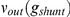 slope, vs. the level of asynchronous release at model synapses. ***E*** Transfer properties in high conductance regime for a model neuron driven by the activity-independent noise (as described in Text) of different intensity: 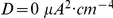 (black triangles); 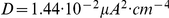 (blue circles); 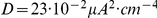 (red squares). In these simulations, 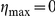. Inset: gain (averaged slope of input-output curve) plotted vs. the intensity of noise. ***F*** Transfer properties in low conductance regime for a model neuron driven by the activity-independent noise of different intensity. Symbols are the same as in ***E***. Inset: gain (averaged slope of input-output curve) plotted vs. the intensity of noise. ***G*** Gains, plotted vs. the output rate, for different scenarios shown in ***E,F***. Top panel: gains for a model neuron in high conductance regime. Bottom panel: gains for a model neuron in low conductance regime. ***H*** Output firing rate, plotted vs. membrane conductance, for a model neuron driven by activity-independent noise. Inset: maximal absolute value of 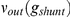 slope, vs. the intensity of noise at model neurons.

**Figure 4:**
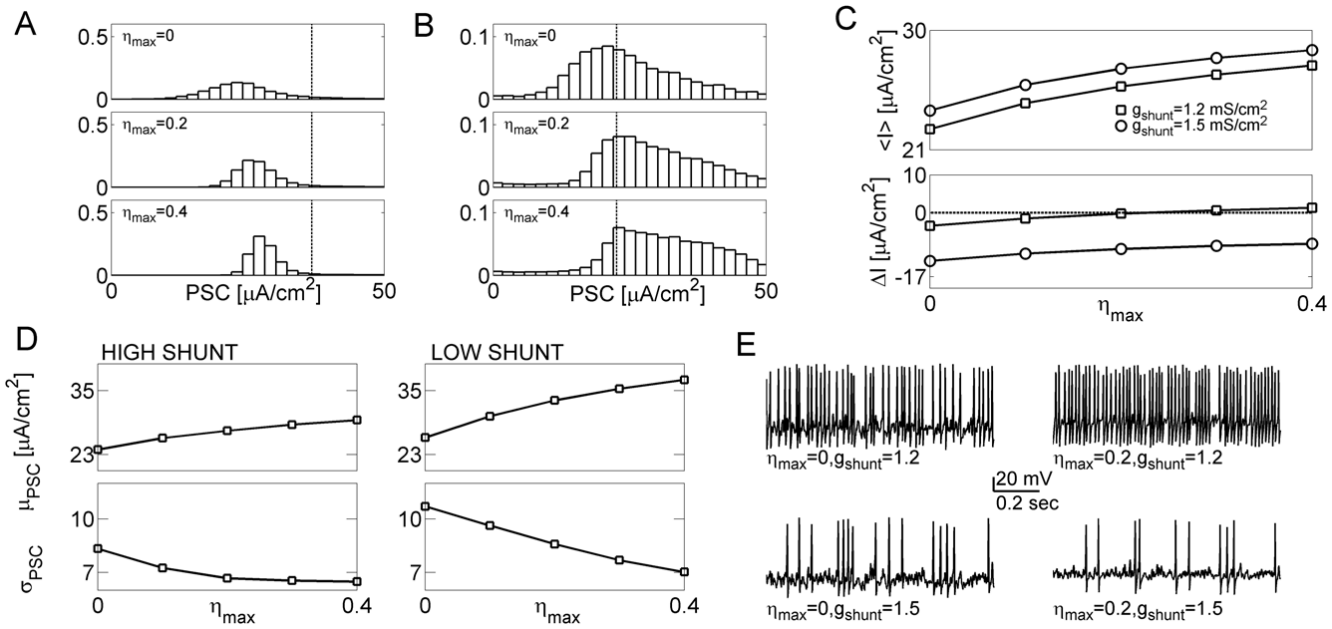
Asynchronous release modulates the statistical properties of post-synaptic current. ***A*** Distributions of postsynaptic current for a model neuron in high conductance regime, and different levels of asynchronous release at model synapses: 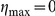 (top); 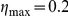 (middle); 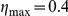 (bottom). Dashed line marks the threshold for spike generation by synaptic-like current of the corresponding magnitude (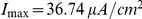) and decay time 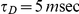. Stimulation rate is 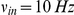. ***B*** Distributions of postsynaptic current for a model neuron in low conductance regime, and different levels of asynchronous release: 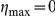 (top); 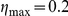 (middle); 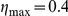 (bottom). Dashed line marks the threshold for spike generation by synaptic-like current of the corresponding magnitude (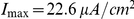) and decay time 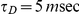. Stimulation rate is 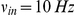. ***C*** Averaged postsynaptic current (top panel) plotted vs. the level of asynchronous release at model synapses. Squares: low conductance regime (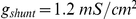). Circles: high conductance regime (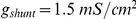). Bottom panel: the difference 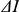 between averaged postsynaptic current and threshold current 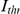. ***D*** Left: mean (top) and standard deviation (bottom) of the postsynaptic current plotted vs. the rate of asynchronous release for a model neuron in high shunt regime (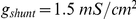). Right: mean 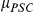 (top) and standard deviation 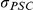 (bottom) of the postsynaptic current plotted vs. the rate of asynchronous release for a model neuron in low shunt regime (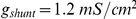). ***E*** Examples of membrane potential. Top left: 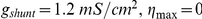. Top right: 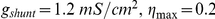. Bottom left: 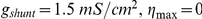. Top right: 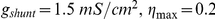.

The effect of shunting on the gain change induced by asynchronous release is shown in Figure 3. In the high shunt regime (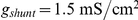, Figure 3A), progressively increasing levels of asynchronous release led to the reduction of both the slope (gain) and the plateau levels of the firing rate curve, making the neuron less sensitive to changes in input rate. For low input rates, the effect of activity-coupled noise due to asynchronous release produced divisive gain modulation. By contrast, when the membrane conductance was in the low shunt regime (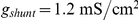, Figure 3B), higher levels of asynchronous release consistently led to an increase in the slope of input-output relation (inset to Figure 3B). A plot of gains vs. output rate, shown in Figure 3C, summarizes the effects of membrane conductance and different asynchronous release rates on the synaptic gain. Figure 3D shows the neuronal firing rate (for fixed input rate 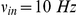) as a function of 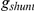, for different levels of 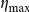. Without asynchronous release, the output firing rate depended almost linearly on the membrane conductance. Increasing levels of asynchronous release sharpened the distinction between high- and low- shunt states, and resulted in distinctive modulation of the neuronal gain (see also insets to Figures 3A,B). Traces of the membrane potential for these different regimes are shown in Figure 4.

The effects of membrane conductance and asynchronous release on the gain curve could be merely a consequence of modulation by noise [3], [4]. Alternatively, they might reflect the competition between the two release modes for the available synaptic resource. To assess the contributions of these two influences, we replaced the noise induced by asynchronous release by a noisy current 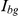 generated by an Orenstein-Uhlenbeck (OU) process with 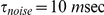 and governed by
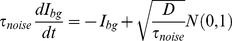
(1)where 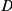 is the intensity of background noise, 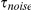 is noise correlation time, and 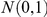 is an uncorrelated Gaussian process with zero mean and unit variance. The resulting input-output curves, shown in Figures 3E,F, suggest that increasing the background noise intensity has a multiplicative effect on neuronal gain in the high shunting regime (Figure 3E), and a weak multiplicative effect for low values of membrane conductance (Figure 3F). The gains for low and high noise levels in the low-shunt regime were almost identical (Figure 3G). With activity-independent “background noise”, the output rate was an almost linear function of membrane conductance (Figure 3H), and the dependence on noise intensity was prominent only in high shunt regime (Figure 3E, compare also to Figure 3D). Note in particular that in the high membrane conductance state, the effect of asynchronous transmitter release was opposite to that of activity-independent “background noise”. High shunting increased the distance to threshold of spike generation by a single brief synaptic stimulus, thus making the neuron able to respond only to relatively strong stimuli (coincidence detector). The mean synaptic current was far below the threshold for spike generation and firing was driven by strong fluctuations in synaptic currents. This is called a fluctuation-driven regime (FDR) [9], [28]. By contrast, in the mean-driven regime (MDR), the rate of spike generation was set by the mean current, whereas fluctuations became relatively insignificant. Because asynchronous release and phasic release draw from the same pool of resource, increasing the rate of asynchronous transmitter release decreased the amplitude of fluctuations due to phasic release; however, the average level of synaptic current was higher. Thus, the effects of asynchronous release on neuronal gain were conditioned by the membrane conductance.

To better understand the effect of shunting conductance on the change in neuronal gain caused by asynchronous release of neurotransmitter, we considered a scenario with more positive leak reversal potential (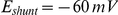 as opposed to 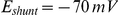 in the baseline model). With a more depolarized leak reversal potential, addition of asynchronous release always resulted in the increase of output firing rate, both for high and low shunt regimes (Supplementary Figure S2A). This is consistent with the notion that in its more depolarized state, the membrane is already in the mean-driven regime where AR acts to increase the output firing rate.

In Figure 4A, the current distribution became more concentrated around its mean with increasing levels of the asynchronous component in the fluctuation-driven regime (also Figure 4C). The distribution of current was above the threshold in the mean-driven regime as shown in Figures 4B,C. In this regime, an increase in the asynchronous component of postsynaptic current (PSC) acted to increase synaptic gain. Thus, asynchronous release has a dual influence that is jointly determined by pre-synaptic activity and the state of the post-synaptic membrane. In Figure 4D the mean and the standard deviation of postsynaptic currents are plotted for different combinations of asynchronous release and membrane conductance. For both high and low conductance states, the mean PSC always increased for higher levels of asynchronous release at model synapses (Figure 4D, top panels). The standard deviation of the PSC decreased with increasing levels of AR at model synapses, for both high and low shunt regimes; however, the standard deviation attained higher values for the model neuron in low conductance state (Figure 4D, bottom panels). Thus, depending on the state of neuronal membrane conductance, asynchronous release of synaptic neurotransmitter differently affected the firing pattern (Figure 4E).

### Competition between phasic and asynchronous releases enables modulation of synaptic gain

Is it possible to modulate the impact of asynchronous release on synaptic gain by controlling the dynamics of synaptic resource availability? We have already seen that in the high shunting regime, variations in the strength of phasic transmission (changing the value of 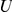) can control the effect of asynchronous release. This occurs when phasic and asynchronous releases compete for the common pool of synaptic resource, and is observed in recordings [12], [16], [23], [24]. However, if phasic and asynchronous modes of synaptic transmission are instead drawn from independent pools of synaptic resource [13], there would follow differential modulation of synaptic gain by the two types of transmitter release.

**Figure 5:**
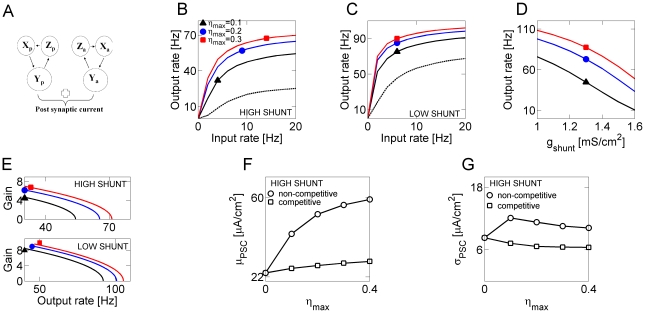
Non-competitive neurotransmitter release dynamics eliminates asynchronous release mediated gain modulation of synaptic stimuli. ***A*** Schematic presentation of non-competitive scheme for dynamics of evoked and asynchronous neurotransmitter releases. ***B*** Transfer properties for high conductance regime (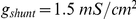) of non-competitive scheme, for different levels of asynchronous release: 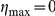 (dashed line); 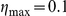 (black triangles); 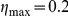 (blue circles); 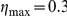 (red squares). ***C*** Transfer properties for a neuron in low conductance regime (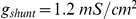). Symbols are the same as in ***B***. ***D*** Output firing rate plotted vs. the membrane conductance, for different levels of asynchronous release. Model synapses were stimulated by Poisson spike trains at 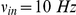. Symbols are the same as in ***B***. ***E*** Gains, plotted vs. the output rate, for high conductance regime (top panel) and low conductance regime (bottom panel) and different levels of asynchronous release (symbols are the same as in ***B,C***). ***F*** Mean postsynaptic current, 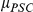, plotted vs. the rate of asynchronous release, for a model neuron in high conductance regime (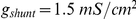). Circles: the “non-competitive” model. Squares: the “competitive” model. Stimulation rate is 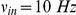. ***G*** Standard deviation of postsynaptic current, 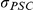, plotted vs. the rate of asynchronous release, for a model neuron in high conductance regime (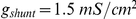). Symbols are the same as in ***F***. Stimulation rate is the same as in ***F***.

To directly assess the effects of competitive neurotransmitter release dynamics in our baseline model, we considered an alternative (non-competitive) synaptic model in which phasic and asynchronous release were decoupled (Equations 10–13 in Methods). Specifically, separate 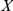 and 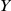 variables were used for the two different release modes (Figure 5A). Analysis of neuronal transfer properties revealed that, in the non-competitive model, the addition of asynchronous release always led to an upward shift in gain curves (Compare Figs. 5B,C,E, with Figures 3A,B,C). These results did not qualitatively depend on the membrane conductance (Compare Figure 5D with Figure 3D), reinforcing the conclusion that the dual effect of 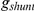 and asynchronous release relied on the competition between the two modes of transmitter release.

An important difference between the competitive and non-competitive forms of asynchronous release was further observed when the mean and the standard deviation of postsynaptic current were plotted against the rate of asynchronous release (Figures 5F,G). In the non-competitive model of asynchronous release, the mean PSC always increased with increasing levels of asynchronous release regardless of the state of neuronal membrane conductance (Figure 5F for high conductance state; low conductance state not shown). For a model neuron in the high-conductance state, the standard deviation of the postsynaptic current in the non-competitive model rose and then decreased with higher levels of asynchronous release (Figure 5G; low conductance state not shown); however, the absolute value of PSC standard deviation was significantly higher for the non-competitive scenario. Thus, the coupling between phasic and asynchronous modes of neurotransmitter release in the model underlies the preferential switch from integration to coincidence detection for different values of membrane conductance.

### Structured afferent activity and asynchronous release differentially modulate synaptic gain

The rate and the variability of neuronal firing can be significantly affected by correlation among afferent inputs [8]. Because phasic and asynchronous release differ in their relative amplitude (high amplitude for phasic release vs. low amplitude for asynchronous release) and time-scale (fast for phasic release vs. slow for asynchronous release), they define distinct temporal windows for signal integration and spike generation [15], which may differentially influence how input correlations affect the neuronal firing patterns.

**Figure 6:**
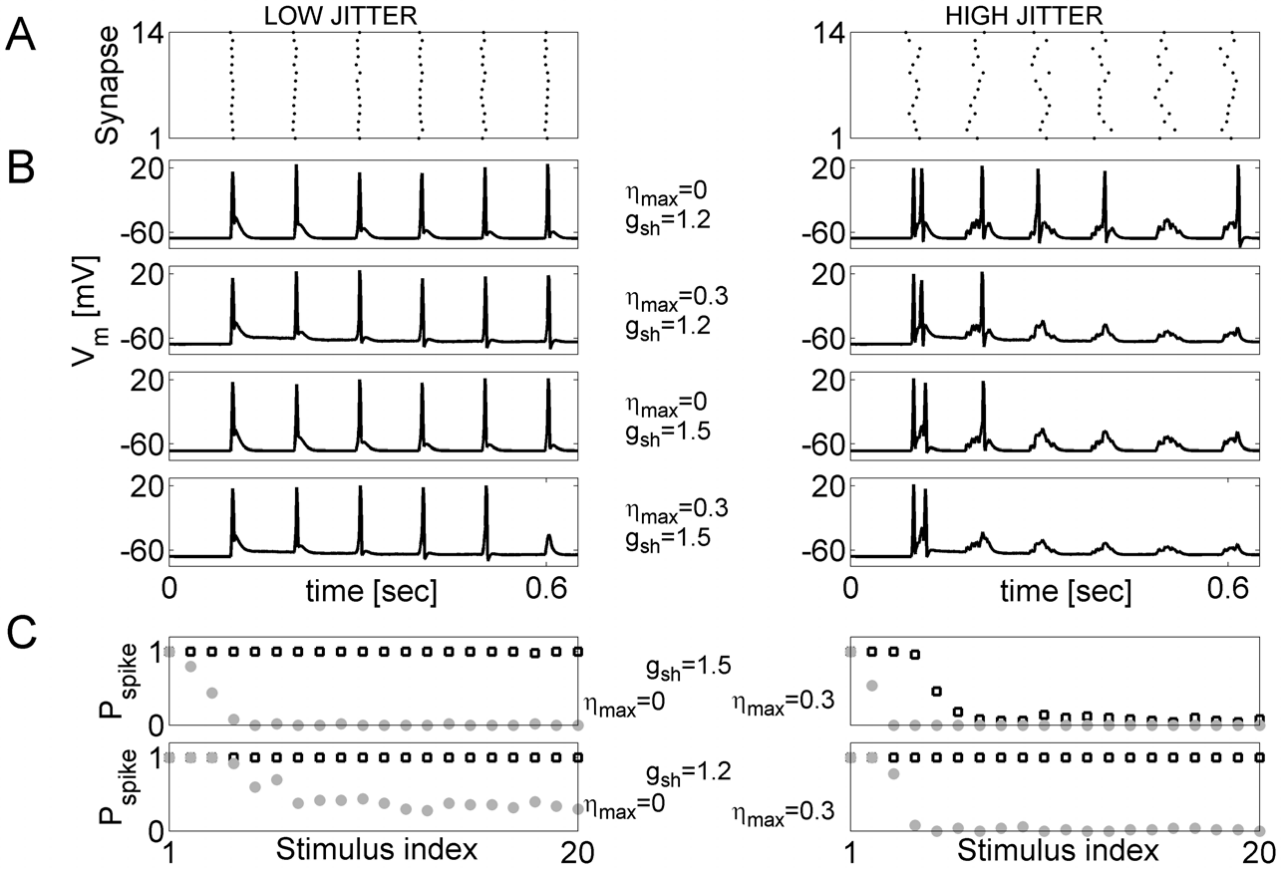
Asynchronous release and membrane conductance define neuronal response sensitivity to correlated input patterns. ***A*** Examples of rhythmic stimulation with controlled level of jitter between the inputs (left panel: 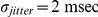; right panel: 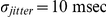, stimulation rate is 10 Hz). ***B*** The effect of asynchronous release (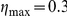, second and fourth panels; 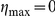, first and third panels) is to decrease the response to later stimuli when the level of jitter is high (left: 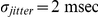; right: 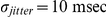, all panels). This effect is more pronounced in high membrane conductance regime (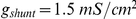, third and fourth panels; 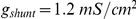, first and second panels). ***C*** The probability to generate spike in response to i-th stimulus (stimulation rate is 10 Hz) for different scenarios. Top left: 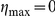, 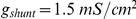; Top right: 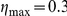, 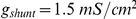; Bottom left: 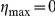, 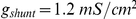; Bottom right: 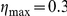, 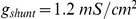. In all panels, open circles are for 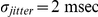, and closed circles are for 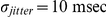. Data points are averages over 50 realizations.

We first considered the response of our baseline model neuron (with competitive dynamics of phasic and asynchronous releases) to a rhythmic and coordinated stimulation of 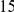 model synapses. The jitter between spikes on different afferents was varied (parameterized by the standard deviation of normal distribution from which the jitter times for individual synaptic channels were drawn) (Figure 6A). Figure 6B shows that in the absence of asynchronous release, neuronal responses to sequences of stimuli were highly reliable with low jitter and low membrane conductance. Adding asynchronous release accentuated the effect of jitter and membrane conductance by making the transition from reliable to unreliable responses sharper (Figure 6C). This is consistent with the previous observation that in the high conductance regime, asynchronous release acted to reduce the gain (Figure 3).

**Figure 7:**
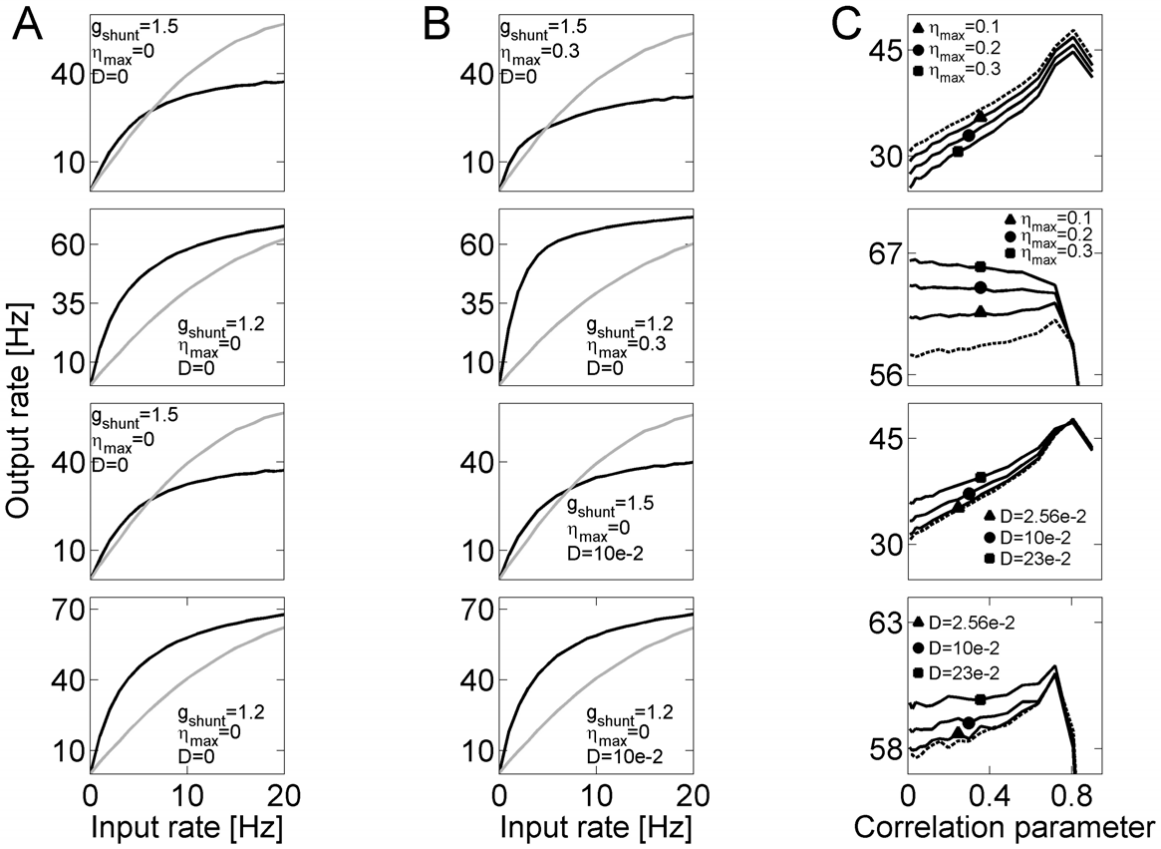
Effects of structured afferent activity and asynchronous release on neuronal transfer properties. ***A*** Top panel: Transfer curves for high shunt (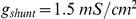), 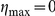, and different levels of afferent correlation: 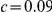 (black), 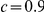 (gray). Second panel: Transfer curves for low shunt (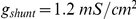), 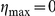, and different levels of afferent correlation: 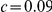 (black), 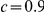 (gray). Third panel: Transfer curves for high shunt (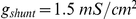), no OU noise (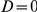), and different levels of afferent correlation: 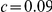 (black), 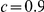 (gray). Fourth panel: Transfer curves for low shunt (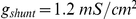), no OU noise (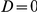), and different levels of afferent correlation: 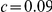 (black), 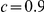 (gray). ***B*** Top panel: Transfer curves for high shunt (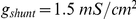), 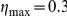, and different levels of afferent correlation: 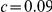 (black), 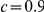 (gray). Second panel: Transfer curves for low shunt (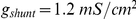), 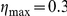, and different levels of afferent correlation: 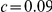 (black), 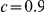 (gray). Third panel: Transfer curves for high shunt (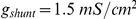), OU noise (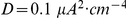), and different levels of afferent correlation: 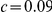 (black), 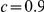 (gray). Fourth panel: Transfer curves for low shunt (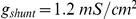), OU noise (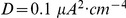), and different levels of afferent correlation: 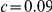 (black), 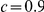 (gray). ***C*** Top panel: Output rate in high shunt (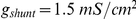) vs. the correlation parameter and different levels of asynchronous release. Dashed line: The case of 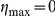. Solid lines: 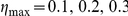. Second panel: Output rate in low shunt (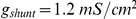) vs. the correlation parameter and different levels of asynchronous release. Dashed line: The case of 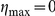. Solid lines: 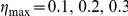. Third panel: Output rate in high shunt (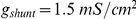) vs. the correlation parameter and different intensities of OU noise. Dashed line: The case of 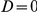. Solid lines: 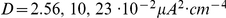. Fourth panel: Output rate in low shunt (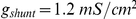) vs. the correlation parameter and different intensities of OU noise. Dashed line: The case of 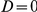. Solid lines: 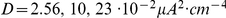. For all cases in ***C*** synapses were stimulated by Poisson spike trains at 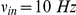.

We generated correlated inputs to the synapses (see Methods) to study the effects of temporally structured non-rhythmic input on the gain properties of a model neuron, with particular emphasis on the modulating action of the slow time-scale, asynchronous, release (Figure 7). In the high shunting regime (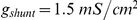, Figure 7A,B top panels), increasing the correlation between afferents led to a generic increase in output rate. Higher transient synchrony between afferents in a fluctuation-driven regime (measured by the correlation parameter, 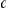) increased the probability that a synaptic input would cross the threshold of spike generation. On the other hand, higher rates of asynchronous release counteracted the effect of correlation by weakening the impact of phasic fluctuations, thus leading to a decrease in neuronal gain (Figure 7C top panel, different curves).

In the low shunting regime (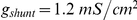, Figure 7A,B,C second panels), the results were reversed. The firing in this regime was driven by the asynchronous component of synaptic current. Grouping inputs together (as a result of correlation) effectively decreased the window for the postsynaptic current, thereby making it more difficult to generate a spike and decreasing the neuronal gain. In contrast to the high-shunt regime, the higher asynchronous release rates increased the firing rate by acting on the asynchronous component of synaptic current.

Consistent with this explanation for the dual role of asynchronous release in shaping neuronal transfer curve, the model neuron driven by an activity-independent noise (Equation 1) always produced higher rates for higher noise intensities (Figure 7, third and fourth panels).

## Discussion

**Figure 8:**
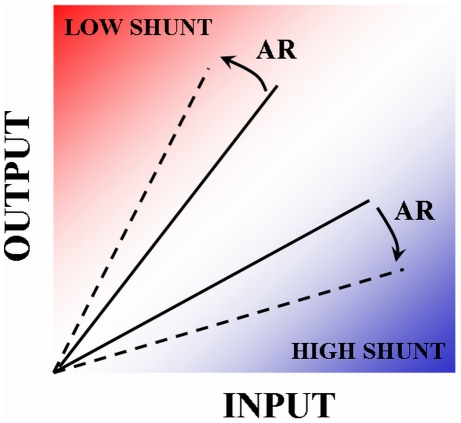
Membrane shunting dependent gain modulation by asynchronous release of neurotransmitter. The action of asynchronous neurotransmitter release on neuronal transfer curve depends on the state of postsynaptic membrane conductance. In high shunt regime, asynchronous release reduces neuronal firing rate. In low shut regime, asynchronous release acts to increase neuronal firing rate.

Sherman and Guillery [29] classify thalamic afferents as either drivers, which are strong and initiate spikes, or modulators, which provide a background context. We have shown here that the same afferent synapse can both drive and modulate depending on the dynamics of presynaptic calcium, which itself depends on the pattern of afferent activity (Figure 8). Thus, drivers and modulators should be considered physiological rather than anatomical concepts.

Neuronal gain can be modulated by a variety of factors. Previous models suggested that shunting inhibition was subtractive [30], but more recent modeling [5] and experimental studies [4] have demonstrated that, in the presence of synaptic excitation, shunting inputs can have a divisive effect on neuronal gain by modulating the slope of input-output curve. We have shown that the membrane conductance can determine the modulating action of activity-coupled asynchronous release of neurotransmitter by switching between divisive and multiplicative modes of action (Figure 8). In addition, we showed that the impact of correlated stimuli on the output firing rate depended jointly (and oppositely) on membrane conductance and asynchronous synaptic transmission. Thus, the postsynaptic membrane conductance, when considered together with presynaptic plasticity and structured input patterns, had an impact beyond that of shifting or scaling the gain curve.

From a computational perspective, the strength of asynchronous release is a single control parameter that controls gain modulation. This is in contrast to other mechanisms of gain modulation, such as balanced background input [3] or concurrent action of shunting inhibition and synaptic excitation [4], which depend on several control parameters. For background input to modulate the gain in divisive manner, the excitatory and inhibitory firing rates have to be increased at the same time [3]. Similarly, divisive gain control by shunting inhibition requires coordination of the latter with synaptic excitation [4]. This poses tight constraints on the dynamics of upstream networks. Asynchronous release affects the gain without additional constraints, and shunting inhibition acts only as a switch that determines the type of the transformation (division or multiplication) performed by the neuron. We showed that the ability to control the gain by single parameter critically depended on the assumption that phasic and asynchronous releases compete for the same pool of synaptic resource [12], [13], [16], [23], [24] (Figures 4,5). Whether or not such competition is a universal feature of central synapses will be resolved by studying the structural organization of these synapses. However, in our model this effect was observed over a relatively narrow range of parameters, suggesting that other mechanisms for gain modulation may also be important.

Recently, it has been shown that the relative strength of phasic and asynchronous releases can be dynamically modulated in hippocampal synapses [31]. These studies showed that selective alteration of asynchrony in transmitter release can be attributed to the protein kinase C (PKC) dependent mechanisms at presynaptic boutons [31]. Our computational model provides an understanding of how modulation of asynchronous release can affect neuronal gain. Taken together with the experimental results reported in [31], our work offers new insight into the principles of computation at the single cell level. Additional experiments aiming to probe changes in synaptic transmission in response to calcium and PKC-modulating second messenger molecules could enhance our understanding of the effects that different modes of neurotransmitter release have on gain control.

What happens in the limit of a large (∼10,000) number of afferent synapses? A simple scaling of synapse number and all relevant parameters would offer a simple way to extrapolate our findings for the simplified “lumped” neuron to this realistic limit (Supplementary Figure S1). However, central neurons often possess highly ramified dendrites with a multitude of active conductances [32]. The dendrites of a pyramidal neuron could in some circumstances behave as independent processing units and the soma could be considered as a second layer in a two-layer artificial neural network, evaluating the results of many converging local dendritic computations [33]. Strong spatial separation of functionally similar (synchronously activated) synapses could compromise the detection of asynchronous release. In addition, localized changes in membrane conductance (for example, due to the local action of inhibition or adaptation) could screen the effect of temporally structured activity and asynchronous release. Both of these problems could in principle be overcome if synapses were distributed on dendritic branches according to their functional similarity, so that the probability of synchronously activated synapses is higher when they are located on the same branch. Although inputs to pyramidal neurons might be spatially organized [33], the effects of synaptic distribution on gain modulation by asynchronous release should be further investigated.

In the point-like neuronal model that we studied here, the membrane conductance was controlled by a single parameter, and the effect of gain modulation by asynchronous release of neurotransmitter was observed over a relatively narrow range of membrane conductance values. However, in real pyramidal neurons, comprised of many dendritic compartments, membrane conductances can vary across different branches and can be modulated locally by a variety of factors including locally acting shunting inhibition due to the inputs from interneurons, and the activation of potassium currents, particularly the slow after-hyperpolarization (sAHP). Considered together with the notion of two-layer network and the existence of asynchronous release, such local modulation of dendritic excitability could provide pyramidal neurons with significant computational capacity. In this view, the pyramidal neuron should be viewed as composed of many dendritic computational units [33], each of which could adapt the nature of its computation (divisive or multiplicative) based on the patterns of ongoing activity. Local dendritic modulation of membrane conductance could also potentially widen the range over which asynchronous release affects gain modulation. More detailed computational models should examine whether this could indeed occur.

The size of the active zone of synapses and their release characteristics are heterogeneous [25], [34]. The ability of an active zone to support asynchronous release might depend on its size. Small active zones contain fewer readily-releasable vesicles, and therefore tend to exhibit low release probabilities, which would render asynchronous release less frequent. Larger synapses can accommodate significantly more vesicles that can be released spontaneously.

Asynchronous release has not been as well studied as phasic release. Asynchronous release onto a dendrite could be screened or boosted, depending on the ion channels in the dendritic tree. Future integrated experimental and modeling studies could resolve the question of how the distribution of dendritic mechanisms affects the impact of synaptic plasticity and different modes of neurotransmitter release in determining neuronal spike discharge patterns.

## Methods

We constructed both a vesicular model of neuronal excitation as well as a simplified approach, which makes use of the coordinated activation of 1% of the synapses impinging on a fixed neuron. The reduced model used here is based on one that was previously matched semi-quantitatively with experimental findings and used to study reverberatory networks in hippocampal cultures [12], [19]. Because we focused here on short-term influences, the present model did not include slow synaptic depression, which is needed to terminate the reverberatory activity in recurrent networks. We modified the neuronal dynamics (following [27]) by including biophysical mechanisms to account for the properties of spike generation in central neurons.

All equations were integrated with custom software written in C, using the second-order Runge-Kutta method with a fixed time step *Δt* = 0.025 msec.

### Neuronal dynamics

We used a conductance-based model with one compartment [27], [35], which is a compromise between a detailed multi-compartmental model that encompass realistic dendritic morphologies and an oversimplified integrate-and-fire model. The ionic current through the model neuronal membrane was taken to be:
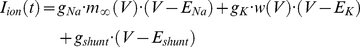
(2)

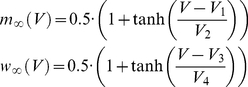
(3)

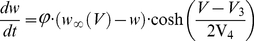
(4)The membrane potential was governed by:
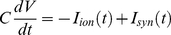
(5)where 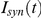 is the synaptic currents discussed in detail in the next section.

The following parameter values were used in the model: 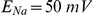, 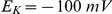, 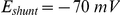, 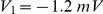, 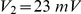, 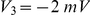, 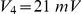, 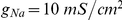, 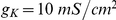, 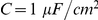, 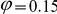. To establish the effect of membrane conductance on neuronal processing of synaptic stimuli, the value of 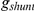 was varied in the range 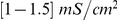. With this choice of parameters, the model neuron exhibited Type-2 excitability, with the transition between quiescent and spiking states described by a Hopf bifurcation [27]. In additional simulations (Text S1) we also investigated the changes that would ensue with Type-1 neuronal dynamics (transition to spiking via saddle-node bifurcation). Results obtained with Type-1 model were qualitatively similar to those reported in Text, reinforcing our assumption that the phenomenon we report is fairly general with respect to the detailed bifurcation structure underlying the neural excitability.

### Vesicular model of synaptic dynamics

The model we used here is based on the classical quantal model of synaptic transmission [36], extended to account for the existence of asynchronous release. We assume that each synaptic connection is composed of N release sites (N = 5). Each site can accommodate at most one vesicle that is available for release, and the release from each of the N sites that constitute the synaptic connection is independent of the release from all other sites. Immediately following the arrival of action potential each site can release its vesicle (if available) with probability 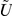 (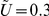). In addition to this phasic release, asynchronous release can occur in the time interval 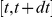 with probability 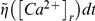 that depends on the level of presynaptic residual calcium -
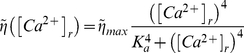
(6)

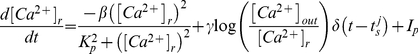
(7)In Equation 6, the rate of asynchronous release depends on the presynaptic concentration of residual calcium, 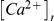, which increases due to action potentials in proportion to the electrochemical gradient across the synaptic membrane, and decays nonlinearly due to extrusion by active calcium pumps. The term 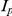 ensures that in the absence of any presynaptic spikes, synaptic calcium is maintained at a non-zero steady-state level (∼50 nM). Once the release of the vesicle from the release site occurs (either in phasic or asynchronous way), the site can be refilled in any time interval 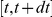 with probability 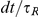.

We typically investigated the neuronal response to the stimulation of 2000 synapses modeled as described above (since each synapse has 5 independent release sites, overall there are 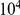 release sites driving the model neuron in this scenario). Collective neuronal activity in early development is characterized by synchronized bursting events, during which most of the recorded neurons are engaged in highly correlated activity [37]. Thus, we assumed that there exists a certain amount of synchrony between the afferents. Specifically, we assumed that the entire set of 2000 synapses is activated by 100 “drivers”, so that every 20 out of 2000 synapses (1%) are synchronously activated.

Synaptic conductance in response to the release (either phasic or asynchronous) of neurotransmitter from presynaptic terminal rose instantaneously and then decayed exponentially
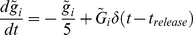
(8)The values of maximal synaptic conductances, 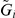, were chosen from a Gaussian distribution truncated at 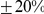 around the mean value 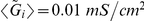.

In the vesicular model, the synaptic current due to the activation of afferent inputs (Equation 5) was
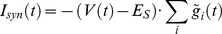
(9)where the sum is over all afferents *i*. For neuronal responses to the stimulation of AMPAergic synapses, the reversal voltage was taken as 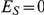. We wanted to focus on the effects of synaptic depression and asynchronous release, and therefore in these studies all of the afferent synapses were excitatory.

### Reduced model of synaptic dynamics

Since long-term simulation studies of vesicular model become intractable in the limit of a large number (thousands) of synapses, in most of our studies we used a phenomenological model that described the synchronous activation of several active zones [19], [38]. In this model, the effective synaptic strength (synaptic resource) is assumed to be shuttling between three states: recovered (X), active (Y), and inactive (Z). The equations that describe the dynamics of the synaptic resource are:
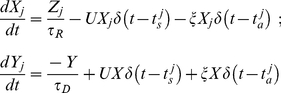
(10)

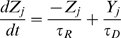
(11)

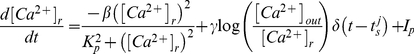
(12)

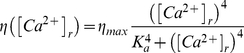
(13)Equations 10–13 describe the response of a *j*-th model synapse to action potentials that occur at times 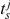. In addition to fast phasic transmission (the strength of which is modeled here as 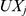), there were asynchronous synaptic events of amplitude 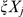 that were generated at times 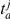. These asynchronous release events were generated by a Poisson process with a time-dependent rate 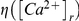. This rate depended on the presynaptic concentration of residual calcium, 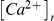, which increased due to action potentials in proportion to the electrochemical gradient across the synaptic membrane, and decayed nonlinearly due to extrusion by active calcium pumps. The term 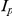 ensured that in the absence of any presynaptic spikes, synaptic calcium was maintained at a non-zero steady-state level (∼50 nM). A schematic diagram of the model for synaptic transmission is shown in Figure 1C.

The firing rate of a neuron depends on the number of inputs and their rate of activation. The number of afferent synapses in real neurons varies across different types of neurons, which for cortical pyramidal neurons it approximately 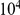. In the reduced model, the number of inputs was taken to be 100 (unless specifically indicated) for the reasons described below; The mean-field equations for synaptic transmission used here represent the ensemble activity of several simultaneously activated active zones. Assuming that an individual active zone contributes ∼0.2 mV to the postsynaptic potential [39] and assuming linear summation of different contributions, the PSP of a single model afferent (∼6 mV) represents activation of ∼30 synapses. This number corresponds to the fraction of synapses that release their vesicles successfully following the arrival of an action potential. In our reduced model this fraction is parameterized by 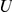, which in most of our studies was assumed to be 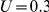. Therefore, the number of synchronously activated synapses is ∼100. Each of the synapses in the reduced model thus corresponds to 100 real synapses and overall the 100 afferents in our reduced model accounted for 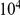 real synapses [40].

The maximal rates of asynchronous release in the reduced model normally ranged from 0 to 3 quanta per millisecond. Keeping in mind that each of the model synapses in the reduced model represents activation of ∼100 real synapses, the rate of asynchronous release considered here would correspond to a maximal rate of ∼0.03 quanta per millisecond for a real single synapse. This is consistent with Goda and Stevens [10] who reported that for synapses established between cultured hippocampal neurons, the rate of asynchronous release at 50 milliseconds following the stimulation could be as high as 0.3 quanta per millisecond. In these studies, the number of activated synapses was not known, but if we assume that a pair of neurons in culture establishes 10 synapses [41] our choice for the rate of AR is consistent with these results [10]. To compensate for the 100-fold higher rate of asynchronous release at our model synapses (as compared with the real single synapses and vesicular release model), the factor 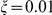 was added to the asynchronous term in Equation 10. The key results pertaining to the dual shunting-dependent effect of asynchronous release that were obtained with the reduced model were also supported by the vesicular model of synaptic transmission (Text S1), thus indicating that the transformation from “discrete” to “ensemble” presentations of synaptic transmission is not crucial.

In the reduced model, the synaptic current due to the activation of afferent inputs (Equation 5) was
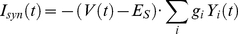
(14)where the sum is over all afferents *i*, and reversal potential 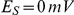. The values of maximal synaptic conductances, 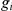, were chosen from a Gaussian distribution truncated at 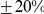 around the mean value 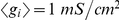.

The following parameter values were used to model the properties of synaptic transmission: 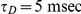, 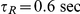, 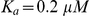, 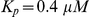, 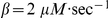, 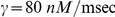, 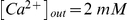, 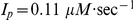, 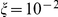.

### Correlated inputs

To generate correlated activity at model afferents, we followed Rudolph and Destexhe [42]. At each time step 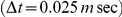 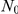 Poisson-distributed events were generated, with 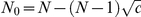. These 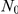 events were then randomly distributed across 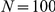 model synaptic channels. The correlation between the activities of different afferents follows because the correlation parameter, 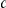, introduces instantaneous redundancy in synaptic activity 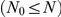.

### Effects of inhibitory afferents

In this study, we focused on the possible effects of synaptic depression and asynchronous release at glutamatergic AMPA synapses. However, significant asynchronous release also occurs at the GABAergic synaptic terminals [11], [18]. The presence of inhibition could in principle attenuate the effects of glutamate asynchronous release on gain modulation. Therefore, in a separate set of simulations, we modeled inputs from both excitatory and inhibitory synapses and considered a variety of possible scenarios in order to test the robustness of our observations with respect to the addition of inhibition. Results of these studies, reported in supplementary material (Text S1 and Supplementary Figure S3), indicate that our qualitative observations regarding the role of asynchronous release in gain modulation do not depend on the presence or absence of input from inhibitory synapses.

## Supporting Information

Figure S1 Effects of AR on input-output transfer properties in the vesicular model of synaptic transmission. ***A*** Transfer curves for high shunt regime (g_shunt_ = 1.5 mS/cm^2^). Black squares: AR rate is η_max_ = 0 quanta/sec. Red circles: AR rate is η_max_ = 6 quanta/sec. Solid lines are linear fits *y = ax+b* to the low-frequency subset of data points (up to input rate of 15 Hz). Fit parameters are *a = 1.14,b = 2.23* (black line) and *a = 0.15,b = 6.3* (red line). ***B*** Transfer curves for low shunt regime (g_shunt_ = 1.2 mS/cm^2^). Symbols are the same as in ***A***.
(0.02 MB EPS)

Figure S2 Effects of neural excitability on asynchronous release modulation of synaptic gain. ***A*** Transfer curves for high (***A1***, g_shunt_ = 1.5 mS/cm^2^) and low (***A2***, g_shunt_ = 1.2 mS/cm^2^) shunt regimes, in the scenario of altered reversal potential of leak current (*E*_L_ = −60 mV). Black squares: η_max_ = 0. Red squares: η_max_ = 0.3. Data points are averages over 20 independent realizations. ***B*** Transfer curves for high (***B1***, g_shunt_ = 1.5 mS/cm^2^) and low (***B2***, g_shunt_ = 1.2 mS/cm^2^) shunt regimes, in the scenario of Type-1 excitability. Black squares: η_max_ = 0. Red squares: η_max_ = 0.3. Data points are averages over 20 independent realizations.
(0.04 MB EPS)

Figure S3 Effects of inhibitory synaptic transmission and asynchronous release on synaptic gain modulation. ***A*** Transfer curves for high shunt (g_shunt_ = 1.5 mS/cm^2^). Closed squares: η_max_ = 0. Open circles: η_max_ = 0.3. ***B*** Transfer curves for low shunt (g_shunt_ = 1.2 mS/cm^2^). Closed squares: η_max_ = 0. Open circles: η_max_ = 0.3. ***C*** Transfer curves for high shunt with selective manipulation of AR at inhibitory synapses. Closed circles: η_max_^IN^ = 0. Open circles: η_max_^IN^ = 0.2. ***D*** Transfer curves for high shunt and different GABA-to-AMPA conductance ratios. Open circles: *R*_I/E_ = 1. Closed circles: *R*_I/E_ = 2. ***E*** Transfer curves for low shunt. Symbols are the same as in ***D***. ***F*** Transfer curves for high shunt, *R*_I/E_ = 2, and selective manipulation of AR at inhibitory synapses. Symbols are the same as in ***C***.
(0.03 MB EPS)

Text S1 Material in this file describes the dependence of AR-mediated gain control on different scenarios: presence of inhibitory inputs, different types of neuronal excitability, different models of synaptic transmitter release.
(0.07 MB DOC)
