# Supplementary material for: Shunting Inhibition Controls the Gain Modulation Mediated by Asynchronous Neurotransmitter Release in Early Development
Source: PLoS Comput Biol. 2010 Nov 4;6(11):e1000973. doi: 10.1371/journal.pcbi.1000973 (PMC2973817; doi:10.1371/journal.pcbi.1000973)
Supplement: Text S1 — Material in this file describes the dependence of AR-mediated gain control on different scenarios: presence of inhibitory inputs, different types of neuronal excitability, different models of synaptic transmitter release. (0.07 MB DOC) [file pcbi.1000973.s004.doc]

**Supplementary Material**

**Gain modulation in vesicular model of synaptic transmission**

Central pyramidal neurons receive input from thousands of synaptic terminals, and the statistical features of neurotransmitter release from these terminals depend on the history of their prior activity. In most of our studies (Text) we employed the “mean-field” description of synaptic transmission, whereby it is assumed that each model synapse represents synchronous activation of a group (usually 100) of biological synapses. This approximation allowed us to significantly reduce the computational load associated with the long-term simulations of a large number of synaptic terminals. Below, we report the results of pilot simulations in which we studied gain control by asynchronous release in a more detail synaptic model that accounted for stochasticity in synaptic transmission and for the large (10 thousands) number of synapses that activate the pyramidal neuron. Input-output transfer curves obtained with this much more realistic model are consistent with the results obtained using a simplified but computationally more efficient phenomenological model (Text), and thus confirm the dual, membrane conductance dependent, role of asynchronous release in gain modulation.

In this more detailed model, the pyramidal neuron is driven by inputs from 2000 afferent neurons. We assume that each afferent synapse has $N_{S}$ release sites ($N_{S}=5$), and at any given time each site can accommodate at most one vesicle. Following the arrival of action potential to the synaptic terminal, each one of the available vesicles can be released (phasic release) with the probability $\tilde{U}$ (we set $\tilde{U}=0.3$ as in the phenomenological ensemble model of synaptic activity). After successful release of vesicle, the release site can be refilled in time interval [t,t+dt] with the probability $dt/\tau_{R}$. In addition to the phasic release, each occupied site can release its vesicle asynchronously with the probability $\tilde{\eta}\left(\left[Ca\right]\right)dt$ that depends on the level of residual pre-synaptic calcium (modeled as described in Methods for phenomenological model of synaptic transmission. Note that $\tilde{\eta}\left(\left[Ca\right]\right)$ is a scaled down version of $\eta\left(\left[Ca\right]\right)$, as used in phenomenological model. To complete the model description, we assume that there exists a certain level of synchrony between the afferent inputs. Specifically, we assume that afferent synapses are activated in groups of 20. Thus, the input to the neuron is defined by 100 uncorrelated Poisson processes, and at any time moment the amount of synchrony between neuronal afferents is ~1 percent.

Figure S1 shows input-output transfer properties obtained for pyramidal neuron driven by the synapses with release properties as described above. The effect of asynchronous neurotransmitter release depends on the level of membrane shunting conductance: in high shunt regime, AR tends to reduce synaptic gain, whereas in low shunt, AR increases the gain. These observations are consistent with the results obtained for phenomenological model of synaptic transmission that assumes averaging over large number of synapses (Methods), and thus make us feel justified in drawing our conclusions based on the results of phenomenological model.

**Effects of leak current reversal potential**

To better understand the effect that various aspects of neural excitability might have on the AR-mediated gain modulation, we here computed the input-output transfer curves for a model neuron with altered value of leak reversal potential (here, we set $E_{L}=-60 mV$ instead of $E_{L}=-70 mV$ as is in the baseline model). Figure S2A shows that with this more positive reversal potential of leak current, the dual effect of AR on synaptic gain disappears – now, addition of AR acts to increase the output firing rate for all input rates under consideration. This is consistent with the notion that more depolarized neuronal membrane favors temporal integration over coincidence detection (and thus mean-driven regime over fluctuation-driven regime).

**Type 1 vs. Type2 models of neural excitability**

Two variable neuronal models are conventionally classified as being either Type-1 or Type-2. The two types differ in the way in which the transition from quiescent state to spiking is accomplished: in Type-1 models it occurs through saddle-node bifurcation, whereas in Type-2 models the transition to spiking is accomplished through Andronov-Hopf bifurcation. The baseline model that we studied corresponds to Type-2 dynamics. We were interested to know whether or not the effect of AR is general enough so that it would exist also in neural models with Type-1 dynamics. Morris-Lecar model studied by us is a good candidate, as it can be easily changed to display Type-1 dynamics. In the model used by us here, the transition from Type-2 to Type-1 is simply made by setting the value of $V_{3}=10 mV$ (instead of $V_{3}=-2 mV$ as is in the baseline Type-2 model). Figure S2B shows that a change from Type-2 to Type-1 dynamics does not qualitatively affect the effect of AR on neuronal gain curves): in high shunt regime, AR still results in the reduction of output firing rate, while it acts to increase the output rate in low shunt regime.

**Effects of synaptic inhibition**

To test the effects of synaptic inhibition, we considered the response of a model neuron driven by inputs from 100 excitatory AMPAergic model synapses and 25 inhibitory GABAergic model synapses. Both excitatory and inhibitory synapses were described by the same model equations, similar to the ones used in the baseline model, as described in the main text. The reversal potential of excitatory synapses was set to $E_{AMPA}$, while the reversal potential of inhibitory synapses was set to $E_{GABAa}$. Other parameters, such as the characteristic recovery time from synaptic depression $\tau_{R}$, the typical time of synaptic conductance decay $\tau_{D}$ and the resource usage parameter, $U$, were the same for both types of synapses.

We considered several scenarios in which we varied the maximal rate of asynchronous release $\eta_{\text{max}}$, the value of the post-synaptic membrane conductance $g_{shunt}$, and the ratio of peak inhibitory to peak excitatory synaptic conductances, $R_{I/E}$. We also considered scenarios in which asynchronous release was selectively added to, or removed from, the inhibitory synapses.

Figure S3A shows that in high conductance regime ($g_{shunt}=1.5 mS/cm^{2}$), higher levels of asynchronous release led to the reduction of firing rate in the manner similar to what is observed in the model without inhibition. However, the effect was less pronounced (compared to the baseline model) in the low conductance regime ($g_{shunt}=1.2 mS/cm^{2}$), as shown in Figure S3B. Selective blockade of asynchronous release at model inhibitory synapses also led to an increase in the firing rate in the manner similar to multiplicative gain change (Figure S3C).

Increasing the ratio $R_{I/E}$ of inhibitory to excitatory peak synaptic conductance led to the general reduction of firing rates (Figures S3D,E,F).
